# Recent Notices of Dr. Gorgas' Treatise on "Dental Medicine."

**Published:** 1884-03

**Authors:** 


AMERICAN JOURNAL

OF

DENTAL SCIENCE.

VOL. XVII. THIRD SERIES—MARCH, 1884. NO. 11

ARTICLE I.

### RECENT NOTICES OF DR. GORGAS' TREATISE ON "DENTAL MEDICINE."

BY C. L. S.

This "DENTAL MEDICINE—A MANUAL OF DENTAL MATERIA MEDICA AND THERAPEUTICS," has met with general favor, and *every* notice which has appeared in the various dental journals (with one exception) have been highly favorable and complimentary to the work and its authur. The exception is a review of this work which appeared in the March number, 1884 of the *Dental Cosmos*, over the initials "A. W. H." whose criticisms led us almost involuntarily to suspect some animus towards Dr. Gorgas, for his objections were almost entirely unfounded, and even had they been true, they were matters of no importance; in fact, so trifling were they, that they amount to a positive, though *unintentional*, compliment to Dr. Gorgas, since it goes to prove, that even in a work of such magnitude, no grave errors are to be found.

Prof. J. Taft in the February number of the *Dental Register* says: "perhaps no one in this country is better able to prepare such a manual than Prof. Gorgas. He has large experience in various directions, that has in an eminent degree qualified him for such a task. His course of instructions as a Professor has, for twenty-five years, embraced the subject of Materia Medica, and in addition to these he has edited the late editions of Harris' Principles and Practice of Dental Surgery, and Harris' Dental Dictionary, and in addition to these he has been editor of the AMERICAN JOURNAL OF DENTAL SCIENCE. The material in these ways gained, has been utilized in the production of this work, one which will be highly prized by theprofession, and especially by dental students who have hitherto had to rely mainly upon text-books, prepared for medical students, upon this branch of study."

Dr. W. C. Barrett, editor of the *Independent Practitioner*, in the March number, says: "Prof. Gorgas has long been known as a ripe scholar, an eminent teacher and a terse and vigorous writer. His long editorial and professional experience conspiciously qualifies him for the preparation of such a work as this, and a brief examination of it convinces the reader that he has not miscalculated his ability. It is a book ot over three hundred pages, enriched with many tables and formularies, with a great deal of untabulated information, the results of the extended experience and studies of the author. It is something more than a Materia Medica, for it contains a chapter on "Diagnosis" that is well worth the price of the book. Such useful information, compressed into almost epigrammatic paragraphs, is peculiarly welcome to practitioners who have not the leisure for extended study, and the book is full of it. We must heartily commend it to every one who is desirous of a complete text book upon dental therapeutics.

The "*New England Journal of Dentistry*," February number, says: "As it comes just as we go to press we cannot now attempt a review. A casual glance, however, convinces us that it is a work of great merit. We can do no better now than to quote in full the modest preface to the work, and reserve for a future number a more extended notice." The editor of one of the most prominent dental

Journals in a letter dated March 17th, 1884, writes to Dr. Gorgas as follows :

"*My Dear Doctor:*—We have delayed noting very fully your 'Dental Medicine' until we could find time to do so good a work approximate justice. Note with surprise 'A. W. H.' in March number of *Cosmos*, and feel a good deal more like criticising *him* than the work itself. In fact I have no *criticisms* to make of the work—It appears to me to be most admirable for the purpose for which it is evidently intended. The remedies 'A. W. H.' refers to as being overlooked, seem to me to be new and untried remedies which it were wise to pass by for the present at least in such a work. I often think that authors must *smile* at the *innocence* if not *ignorance* displayed by those who assume to write a *critical* 'review' of works treating of subjects upon which they really know so little."

Very truly,

---

Dr. George Watt, editor of *Ohio State Journal of Dental Science*, in the March number, writes as follows: "The long experience of Prof. Gorgas in the Baltimore College, and later in the University of Maryland, gives good reason to hope that the right man has undertaken the task. The value of the book is greatly enhanced by a very complete general index. In short, both author and publishers have done their duty, and we cordially commend the work to our readers, without, however, settihg up the claim that it is perfect, for 'to err is human,' and the author himself will doubtless see points for possible improvement."

The editor of the *Southern Dental Journal*, Dr. B. H. Catching, in the March number, says: "This work was received just on the eve of our going to press, which gives us a good excuse for using the author's preface in introducing it to our readers. We will say, however, that with only a hurried glance we are more than pleased with the publication. The name of the author is a guarantee of its worth to the dental practitioners. American dentistry, as well as being intensely practical is producing publications on Dental Surgery worthy of the name. A wide gap was open, however, until this work appeared, which we feel sure will meet the demands of the most exacting.

Dr. Charles E. Pike, editor of the *Dental Practitioner*, in the March number, says: "A work of over three hundred pages, containing such a fund of information systematically and conveniently arranged, that it cannot fail to be recognized as the *best* book of the kind with which the dental profession has been favored. The world-wide reputation of Prof. Gorgas as a successful practitioner, teacher and writer, is a sufficient guarantee that the information conveyed is *reliable*, a most desirable feature in any text-book. After a 'Definition of Subjects' and some 'General Remarks,' the author presents a most valuable chapter on 'Important Points in Diagnosing Affections in the Mouth,' which will prove of incalculable advantage to the inexperienced, and will, no doubt, often help 'older heads' to arrive at correct conclusions in some obscure cases. After enumerating other points, the same writer adds: 'We have not attempted to enumerate *all* the good things in the book, but sufficient to indicate its general character. The need of such a work has long been felt by the profession, and we make no doubt ts rapid sale will express their approval of it." In addition to the above the President and Committee of the Birmingham, England, Medical Institute, through D. C. Lloyd Morgan and Robert Lacendly, Honorable Librarians, have requested the favor of a copy of Dr. Gorgas' "Dental Medicine," for deposit in the Library.

In regard to the criticism in the *Dental Cosmos*, the only dental publication in which an adverse opinion is expressed concerning this work, even its author, who is not the editor, says: "The work of Dr. Gorgas, may be said to be the first elaborate attempt to supply a want which every pracitione r of dentistry has experienced. It contains a large amount of useful information, which is not to be found in any other volume." This critic finds fault with the chapter on

"Important Points in Diagnosing Affections of the Mouth." which he characterizes as calculated to mislead, being wanting in accuracy and incomplete in detail.

So far as the latter charge is concerned, this work was evidently not intended to be a treatise on dental surgery, and the author's intention was to *briefly* refer to the important salient points only in the different affections referred to; concerning the charge of inaccuracy, the writer takes issue with the critic, who is evidently either ignorant or designedly insincere; for example he says and denies that "a recession of the gums and absorption of alveolar processes with a tendency to hemorrhage" occurs in the disease known as "pyorrhea alveolaris." The experience of the writer has been that no case of this affection has ever come under his notice in which there was not "recession of the gums and absorption of alveolar processes," for he considers the formation of "pus pockets" to be characteristic of this disease.

Dr. W. C. Barrett in his review of Dr. Gorgas' work, which we have quoted from the *Independent Practitioner* of March, 1884, says of this same chapter on "Diagnosis" "that it is worth the price of the book;" and Dr. Charles E. Pike in the *Dental Practitioner*, also referring to this same chapter, says: "The author presents a most valuable chapter on "Important Points in Diagnosing Affections of the Mouth, which will prove of incalculable advantage to the inexperienced, and will, no doubt, often help 'older heads' to arrive at correct conclusions in some obscure cases." Here is a difference of opinion between two eminent and well known gentlemen, and an obscure and evidently ignorant, or at least prejudiced, *critic*. In regard to the objection made by this *critic* that various valuable agents have not received attention," the answer may be found in the letter, given above when he says; "The remedies 'A. W. H.' refers to as being overlooked, seem to me to be new and *untried* remedies which it were wise to pass by for the present at least in such a work."

Dr. James W. White in his review of the last edition of Dr. Stockens' work remarked that it contained too much concerning agents not in general use, or words to that effect, His delegated *critic*, however, evidently does not hold a similar opinion, but would introduce a mass of useless matter when the object of the author has been to omit, as he remarks in his preface, all that is not applicable to dental practice, and that will not be of benefit to the dental student. No doubt this *critic* thinks that nothing good emanates from any other than one particular source.

